# Epicardial Adipose Tissue: A Piece of The Puzzle in Pediatric Hypertension

**DOI:** 10.3390/jcm12062192

**Published:** 2023-03-12

**Authors:** Nina Schweighofer, Mitja Rupreht, Nataša Marčun Varda, Primož Caf, Petra Povalej Bržan, Vojko Kanič

**Affiliations:** 1 Department of Radiology, University Medical Centre Maribor, Ljubljanska Ulica 5, 2000 Maribor, Slovenia; 2 Medical Faculty, University of Maribor, Taborska 8, 2000 Maribor, Slovenia; 3 Department of Pediatrics, University Medical Centre Maribor, Ljubljanska Ulica 5, 2000 Maribor, Slovenia; 4 Faculty of Electrical Engineering and Computer Science, Koroska cesta 46, 2000 Maribor, Slovenia; 5 Department of Cardiology, University Medical Centre Maribor, Ljubljanska Ulica 5, 2000 Maribor, Slovenia

**Keywords:** epicardial adipose tissue, hypertension, children, adolescents, magnetic resonance imaging

## Abstract

Background and purpose: Epicardial adipose tissue (EAT) is a metabolically active tissue located on the surface of the myocardium, which might have a potential impact on cardiac function and morphology. The aim of this study was to evaluate whether EAT is associated with essential arterial hypertension (AH) in children and adolescents. Methods: Prospective cardiovascular magnetic resonance (CMR) study and clinical evaluation were performed on 72 children, 36 of whom were diagnosed with essential AH, and the other 36 were healthy controls. The two groups were compared in volume and thickness of EAT, end-diastolic volume, end-systolic volume, stroke volume, left ventricular (LV) ejection fraction, average heart mass, average LV myocardial thickness, peak filling rate, peak filling time and clinical parameters. Results: Hypertensive patients have a higher volume (16.5 ± 1.9 cm^3^ and 10.9 ± 1.5 cm^3^ (*t* = −13.815, *p* < 0.001)) and thickness (0.8 ± 0.3 cm and 0.4 ± 0.1 cm, (U = 65.5, *p* < 0.001)) of EAT compared to their healthy peers. The volume of EAT might be a potential predictor of AH in children. Conclusions: Our study indicates that the volume of EAT is closely associated with hypertension in children and adolescents.

## 1. Introduction

Arterial hypertension (AH) is both a chronic health condition, with persistent high blood pressure, and a global health issue [1]. AH is a major risk factor for adverse cardiovascular outcomes, end-stage renal disease, along with mortality [2]. In recent years, the prevalence of AH in the pediatric population has been rapidly increasing, especially in light of the growing childhood obesity epidemic [3]. The prevalence rate is estimated at 9.7%, which makes AH one of the most common health conditions in childhood [4]. Primary hypertension, or essential hypertension (EH), is defined as elevated blood pressure, with values higher than the 95th percentile for age-, sex- and height-specific percentile tables, without a known cause [5].

Epidemiologic studies propose a link between pediatric AH, subclinical atherosclerosis and target organ damage, especially in terms of the heart [2]. The heart is, therefore, a potential target organ which needs to be evaluated in terms of pediatric AH. Studies have proposed associations between AH, left ventricular ejection fraction and peak global longitudinal strain [6,7]. Heart function is routinely evaluated by ultrasound. However, cardiac magnetic resonance (CMR) is the gold standard imaging modality for evaluating function, with the main drawback being cost-effectiveness [8]. Moreover, CMR offers the possibility to analyze soft tissues surrounding the heart, such as the epicardial adipose tissue (EAT) [9].

EAT is a metabolically active adipose tissue depot located directly on the surface of the myocardium and surrounded by the visceral layer of the pericardium [10]. It covers up to 80% of the myocardium, mainly the right ventricular surface and the anterior wall of the left ventricle. EAT has great elasticity and compressibility and, therefore, protects coronary arteries from mechanical stress caused by artery pulse and myocardial contraction [11]. EAT is a part of the visceral adipose tissue compartment. The main difference between EAT and other visceral fat depots is its greater capacity for the release and uptake of free fatty acids and a lower rate of glucose utilization. EAT acts as an energy source for the myocardium and plays an important role in providing energy to the cardiomyocytes in times of high demand [12,13].

EAT secretes anti-inflammatory and pro-inflammatory adipokines and has a potential impact on cardiac function and morphology [14]. Atherosclerosis has long been recognized as an inflammatory disease. There is growing evidence to conclude that EAT, as a visceral fat depot, may contribute to the pathological process of coronary artery disease (CAD) [15]. Hypertensive adult patients tend to present a higher EAT thickness surrounding the right ventricular wall [16]. Several studies have proposed a positive correlation between body mass index (BMI) and the volume or thickness of EAT, linking obesity with the pathologic inflammatory effects of EAT [17,18,19].

To our knowledge, no studies have examined the associations between the amount of EAT and AH in children and adolescents with normal BMI using CMR. This link could indicate pathological mechanisms as well as the future burden of adverse cardiovascular events in adult life, which could begin in childhood and early adolescence, even in non-obese patients with AH. Therefore, the goal of the study was to determine whether hypertensive pediatric patients with normal BMI have a higher amount of EAT compared to healthy controls utilizing the CMR. Additionally, we separately measured the volume and thickness of EAT in each patient with the intent of determining the interrelation between the two measurements. 

## 2. Materials and Methods

### 2.1. Study Population

We conducted a prospective case-control study which took place at the University Medical Centre Maribor from November 2021 until April 2022; 72 children and adolescents with normal BMI were enrolled. The first hypertensive group consisted of 36 subjects who had been diagnosed with essential arterial hypertension, with values of systolic blood pressure over the 95th percentile for age, sex and height according to the European Society of Hypertension guidelines for the management of high blood pressure in children and adolescents (2016) [20]. The hypertensive patients were treated in compliance with the European Society of Hypertension guidelines for the management of high blood pressure in children and adolescents [20]. The second group was a healthy control group, with normotensive children and adolescents with no known family history and risk factors for cardiovascular disease, which matched the hypertensive group in average age, sex and BMI. The children and adolescents were volunteers invited in collaboration with primary health care pediatricians. The children and adolescents never had an elevated BP measured at the systematic clinical evaluation. The two groups were compared.

### 2.2. Clinical Assessment

Height, weight, waist circumference and hip circumference were measured in both groups. BMI and BSA (body surface area) were calculated. Resting blood pressure was measured in both groups at approximately the same time in the morning hours. Three readings were taken 5 min apart while seated for at least 10 min; the average value was calculated for further analysis. Blood pressure was determined by the same automatic blood pressure monitor (Omron M2, Omron Healthcare, Kyoto, Japan) using an appropriately sized cuff. 

In patients, AH was defined as an average systolic or diastolic BP greater than the 95th percentile according to the age, gender and height of the patient. Detailed percentile tables are presented in the reference article [20]. The measurements were recorded over at least three separate occasions. In cases of hypertension, the diagnosis was confirmed by ambulatory blood pressure monitoring (ABPM) and automatic blood pressure monitoring every 30 min over a 24 h period. The blood pressure values published by Soergel et al. were used as the reference values for ambulatory blood pressure monitoring [21]. AH was diagnosed after a detailed clinical and laboratory diagnostic work-up that closely followed the recently published recommendations outlined in the European Society of Hypertension guidelines for the management of high blood pressure in children and adolescents [20]. Exclusion criteria were identified secondary causes for AH (congenital adrenal hyperplasia, pheochromocytoma, primary hyperaldosteronism, thyrotoxicosis, renovascular hypertension and aortic coarctation) and absolute and relative contraindications for CMR. The diagnosis of essential AH was obtained at the Department of Pediatric Nephrology of University Medical Centre Maribor, where all of the patients were also followed-up and treated.

### 2.3. Imaging Acquisition

CMR was conducted on weekends in the morning hours. Participants fasted for at least 6 h and were well hydrated. CMR was performed on a 1,5 T MR system (Magnetom Sola; Siemens AG, Erlangen, Germany) using an 18-element body coil and a 32-element spine coil. Scout images in coronal sagittal and axial planes were obtained for the planning of the final double oblique long-axis and short-axis views. For morphological measurements, a stack of 8–12 short-axis images, depending on the size of the heart, was planned to cover both the right and left ventricles, including every visual part of EAT. Scans were taken in retrospective mode during repeated eight end-expiratory breath-holds. To evaluate functional parameters, ECG-gated standard two and four-chamber steady-state free-precession (SSFP) images and a short-axis stack of cine SSFP images were acquired. Imaging parameters were as follows: time of repetition 6.7 ms, time to echo 1.12 ms, temporal resolution 35 ms, slice thickness 8 mm, interslice gap 2 mm, flip angle 54°. Total imaging time was 13 min and 16 s, and all of the subjects completed the examination.

### 2.4. Image Analysis

#### 2.4.1. Assessment of the EAT

**Figure 1:**
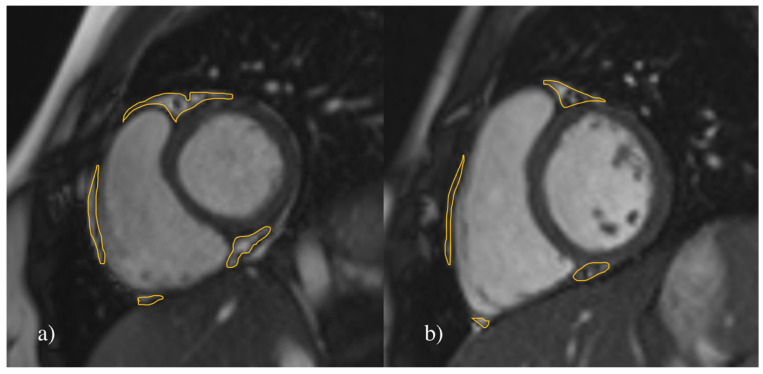
Measurement of the EAT volume. Representative MR short-axis image of the heart in cine SSFP sequence in 17-year-old females. Delineated areas demonstrate epicardial adipose tissue (EAT). The total measured EAT volume in (**a**) a hypertensive patient was 17.62 cm^3^ and in (**b**) a healthy control was 10.35 cm^3^.

**Figure 2:**
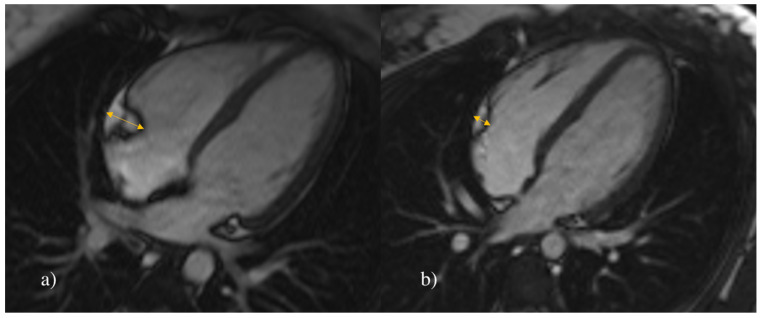
Measurement of EAT thickness. Representative MR four-chamber long-axis image of the heart in cine SSFP sequence in 17-year-old females. Arrows are indicating epicardial adipose (EAT) tissue thickness in right atrioventricular groove. The measured EAT thickness in (**a**) a hypertensive patient was 1.12 cm and in (**b**) a healthy control was 0.44 cm.

The volume of EAT was calculated using Syngo.via imaging software (Siemens AG, Erlangen, Germany). From the morphological stack of images, the contours of EAT were manually outlined in end-diastole in every short-axis slice (Figure 1). The area outlined in each manual tracing was automatically multiplied by slice thickness to yield total fat volume expressed in cm^3^. We additionally measured EAT thickness in an end-diastole four-chamber long-axis image in the right atrioventricular (AV) groove (Figure 2). This location was determined with compliance to previous studies stating that it is the most evident anatomic part for measuring EAT thickness, which can also be easily determined with ultrasound examination [9,22]. 

Functional analysis was also performed using the Syngo.via post-processing software. Each image stack was examined for abnormalities in the morphology of the heart. End-diastolic volume, end-systolic volume, stroke volume, left ventricular ejection fraction, average heart mass, average LV myocardial thickness, peak filling rate and peak filling time were analyzed and indexed according to BSA.

#### 2.4.2. Reproducibility

Measurements of EAT volume and thickness were performed by two radiologists blinded to the groups within a time frame of one week with the intention to establish intra-observer reproducibility. 

### 2.5. Statistical Analysis

The data are presented as mean value (MV) ± standard deviation (SD) or median (M) with interquartile range (IQR) in case of non-normal distribution. BMI was calculated by the following formula: BMI (kg/m^2^) = weight (kg)/height (m)^2^. Body surface area (BSA) was calculated by a variation of the DuBois and DuBois formula: BSA (m^2^) = 0.007184 × [weight (kg)^0.425^ × height (cm)^0.725^] [23]. 

An intraclass correlation coefficient (ICC) was calculated using a two-way mixed effects model as a measure of intra-observer reproducibility for EAT volume and EAT thickness. Analysis of variance (ANOVA) was used to calculate the ICC using a model that treats raters as a fixed factor. ICC values were defined as indicating poor (<0.5), moderate (0.5–0.75), good (0.75–0.90) and excellent reproducibility (>0.90) [24]. Data distribution was determined using the Shapiro–Wilk normality test. An unpaired, 2-tailed Student’s *t*-test and nonparametric Mann–Whitney U test were used to compare the normotensive and hypertensive groups according to whether the data followed a normal distribution. Sex differences were determined using the Fisher’s exact test. A nonparametric Spearman’s Rho test and linear regression analysis were performed on anthropometric, functional and morphologic parameters to identify correlates of EAT thickness and EAT volume between groups. The estimation of the best variable for predicting hypertension was also evaluated with the receiver operating characteristic curve (ROC) and the area under the ROC curve (AUC). The Youden index was used to define a single optimal threshold maximizing the average of sensitivity and specificity. In addition to sensitivity and specificity, the positive predictive value (PPV) and negative predictive value (NPV) were also calculated. Logistic regression was also used to predict hypertension from EAT volume. Multivariate linear regression analysis was used to evaluate whether EAT thickness was an independent predictor of EAT volume. The Akaike information criterion (AIC) was used to estimate the prediction error and, thereby, the relative quality of our statistical models. A value of *p* < 0.05 was considered statistically significant. Statistical analysis was performed using the R programming language (2022, Vienna) [25].

## 3. Results

### 3.1. Study Population, Clinical Assessment and Body Composition

The prospective controlled magnetic resonance study included 72 Caucasian children and adolescents (52 males and 20 females) aged 12–19 years with normal BMI (18.5–24.9 kg/m^2^). The first group consisted of 36 hypertensive patients (27 (75.0%) males, 9 (25.0%) females, aged 15.2 ± 1.72 years). The second group consisted of 36 normotensive subjects (25 (69.4%) males, 11 (30.6%) females, aged 15.3 ± 2.2 years). The groups had comparable BMI and BSA. The details are presented in Table 1. 

### 3.2. Parameters of Cardiac Morphology and Function

Subjects in the normotensive control group and the hypertensive group were compared in parameters of cardiac morphology and function. EAT volume was significantly larger in the hypertensive group compared to the normotensive group (16.5 ± 1.9 cm^3^ and 10.9 ± 1.5 cm^3^ (*t* = −13.815, *p* < 0.001)). EAT thickness was also significantly higher in the hypertensive group compared to the normotensive group (0.8 (0.3) cm and 0.4 (0.1) cm, (U = 65.5, *p* < 0.001)). There were no differences in other parameters (Table 2).

### 3.3. Intra-Observer Reproducibility

Overall, intra-observer reproducibility of EAT volume and EAT thickness measurements using ICC for EAT volume was 0.91 (95% CI, 0.86–0.94) and for EAT thickness was 0.79 (95% CI, 0.67–0.87), indicating good (EAT thickness) to excellent (EAT volume) reproducibility.

### 3.4. Associations of EAT Volume with Anthropometric, Morphologic and Functional Parameters 

There was a positive correlation between EAT volume and body weight (r = 0.48, *p* < 0.0001), EAT volume and BSA (r = 0.49, *p* < 0.0001), EAT volume and diastolic BP (r = 0.55, *p* < 0.0001) and EAT volume and systolic BP (r = 0.85, *p* < 0.0001). No other significant correlation was noted. 

### 3.5. Predicting Hypertension on the Basis of EAT Volume and EAT Thickness 

**Figure 3:**
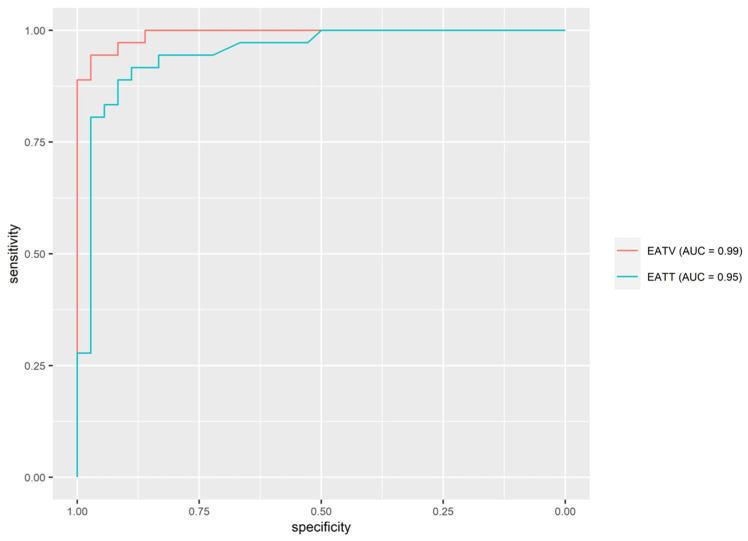
ROC curves and AUC for potential predictors of hypertension. EATT: epicardial adipose tissue thickness; EATV: epicardial adipose tissue volume.

The estimation of the best prediction variable was evaluated with ROC curves and AUC. Out of all selected variables that showed as significantly important in discriminating between the hypertensive and normotensive groups, EAT volume turned out to be the best predictor of hypertension (AUC = 0.99 (95% CI, 0.98–0.99)) with a sensitivity of 94.4% and specificity of 97.2%. Positive predictive value (PPV) and negative predictive value (NPV) were 97.1% and 94.6% (Figure 3). Table 3 shows the AUC, sensitivity, specificity, PPV and NPV, and thresholds for predictors of hypertension.

Additionally, binary logistic regression analysis was performed to predict the probability of hypertension depending on the volume of EAT. Our model showed that increasing EAT volume by one unit results in a 9.5 times higher probability of developing hypertension.

### 3.6. Predicting EAT Volume on the Basis of EAT Thickness

Linear logistic regression was used to evaluate whether EAT volume can be predicted from EAT thickness. Two different models were used. The first model only includes two variables, of which EAT volume is dependent on EAT thickness. Model 1 can account for 64% of the variability in EAT volume (adjusted R^2^ = 0.64, AIC = 306.4). Model 2 includes five independent variables and is adjusted for age, gender, BSA and hypertension. Model 2 is even more accurate and can account for 87% of the variability in EAT volume (Adjusted R^2^ = 0.879, AIC = 236.9). 

## 4. Discussion

The pathophysiologic process of primary AH is extremely complex and cannot be explained solely on the basis of EAT; however, EAT does appear to be a piece of the puzzle. To the best of our knowledge, the association between the volume of EAT and essential AH in children has not yet been established on the basis of CMR. Our major findings were: (1) hypertensive children and adolescents had higher volume and thickness of EAT, (2) the volume of EAT had a positive correlation with body weight, BSA, diastolic BP and systolic BP, (3) EAT volume had a better association with hypertension in comparison with EAT thickness, (4) EAT volume is associated with EAT thickness.

Many studies agree that the amount of EAT is higher in hypertensive adult patients [16,26,27,28,29]. There are fewer studies which propose this link in children and adolescents [19]. The main advantage of our study compared to previously published work is the precise quantification of EAT volume with the aid of the semi-automated CMR method. CMR with post-processing automated or semi-automated volumetry is currently the gold standard tool for evaluating EAT in children [9,30,31]. A vast majority of similar studies did not quantify the volume of EAT but used thickness measurements of EAT assessed by ultrasound examination, which is less reliable and more dependent on the examiner [17,18]. 

The second important difference is the selection of patients; we only choose non-obese children and adolescents. We tried to correlate the amount of EAT and hypertension in healthy-weight children because several studies have already proposed a link between the amount of EAT and hypertension in obese children [17,19,32]. We can conclude the volume of EAT is a potential risk factor even in non-obese children and could be evaluated in clinical practice. 

The third advantage is simultaneously measuring the volume of EAT and thickness of EAT. To our knowledge, this had not been performed in any of the previous studies. We intended to find a superior parameter to predict hypertension in children and came to the conclusion that both the volume and thickness of EAT show a good correlation with hypertension and its severity, but the volume of EAT has a higher predictive value compared to the thickness of EAT. 

Our study showed a strong association between essential AH and EAT in children as young as 12 years old, which raises the question of whether the amount of EAT could be partly genetically predisposed [33]. The exact pathophysiologic pathway of EAT in cardiovascular disease remains uncertain. There are some theories connecting EAT and essential AH. One suggests that EAT is the cause of hypertension. In larger EAT depots, there is an oxygen shortage. On account of that, EAT is invaded by macrophages and lymphocyte T cells, which results in a change in its metabolic profile. The increased volume of EAT then results in higher amounts of secreted pro-inflammatory cytokines and vasoactive peptides, including (IL)-1β, -6, -8 and -10, TNF-α, MCP-1, adiponectin, leptin and angiotensin II. These molecules can later trigger the renin-angiotensin system and cause an increase in arterial blood pressure and hypertrophy of the left ventricle. An increase in plasma-free fatty acid levels may contribute to the stimulation of the autonomic nervous system and subsequently cause an increase in arterial blood pressure [12,34,35]. Another possible theory hypothesizes that AH might determine the build-up of EAT because of mechanisms adapting to higher myocardial energy demands. Long-term effects of AH could cause hyperplasia of cardiomyocytes and capillary proliferation, which subsequently increases the thickness of the myocardium. Rising energy demands could then stimulate EAT accumulation to provide more energy supply with FFA. Some studies have shown that EAT mass increases along with myocardial mass, but the most important is the mass of the left ventricle [36]. Our study is more supportive of the first theory and points in the direction of EAT being one of the risk factors for developing essential AH. We would like to additionally emphasize the fact that there was no significant difference in the average myocardial thickness of the LV nor in the average myocardial mass between groups, which makes us question the second theory. 

The potential clinical application of our study is utilizing the volume of EAT as a prognostic tool in assessing cardiovascular risk in childhood and adolescence. Our study suggests that children and adolescents with a higher volume of EAT have a greater possibility of developing AH. The usefulness of EAT as a possible cardiovascular risk factor can be the identification of the disease at a subclinical stage, preventing future progression through preventive and even therapeutic measures.

It should be noted that our analysis only shows the association between EAT and hypertension and does not establish a causal relationship. Possible pathomechanisms are unclear and, in this study, we did not attempt to investigate the mechanism behind this phenomenon, but we did propose several hypotheses. Further research is needed to determine the pathophysiological effect of EAT on the natural course of hypertension in children and adolescents.

## 5. Conclusions

Children and adolescents diagnosed with AH had a higher volume and thickness of EAT compared to their healthy peers. A better association was found between EAT volume and hypertension compared with EAT thickness and hypertension. Whether EAT can predict hypertension needs to be clarified in future studies.

## 6. Limitations

One limitation of our study is the relatively small number of participants. The second limitation is that our study only included Caucasian children and adolescents with normal BMI; therefore, the generalizability of our results is questionable. Further studies with larger cohorts, including children with different BMI and different ethnic groups, should be considered for providing cut-off values of EAT thickness and volume in terms of pediatric hypertension. The third limitation is that we did not collect blood samples from the entire group of enrolled subjects to measure lipids, pro-inflammatory cytokines or insulin resistance. It is possible that hypertensive children and adolescents have increased pro-inflammatory cytokine signaling, which could mediate and alter the proliferation of EAT [12]. Future studies should measure pro-inflammatory cytokines to clarify the impact that pro-inflammatory cytokines and their targets may have on the metabolism of EAT.

## Figures and Tables

**Table 1:** Anthropometric and body composition parameters of the study population.

	All (N = 72)		Hypertensive (N = 36)		Normotensive (N = 36)		
Variable	MV ± SD	M (IQR)	MV ± SD	M (IQR)	MV ± SD	M (IQR)	*t*/U Value (*p*-Value)
Age (years)	15.3 ± 2	15 (3)	15.2 ± 1.7	15 (2.2)	15.3 ± 2.2	15 (3.2)	U = 649 (0.995)
Height (cm)	174.4 ± 11	172.5 (16.2)	176.8 ± 11.1	175 (10.5)	172 ± 10.5	170 (15)	*t* = −1.877 (0.065)
Weight (kg)	65.9 ± 10.9	66.5 (12.5)	67.8 ± 9.8	70 (15)	64 ± 11.7	60.5 (16.2)	*t* = −1.476 (0.145)
BMI (kg/m^2^)	21.5 ± 2	21.9 (2.8)	21.6 ± 1.6	21.9 (2.4)	21.5 ± 2.3	21.7 (3.9)	*t* = −0.197 (0.844)
BSA (m^2^)	1.8 ± 0.2	1.8 (0.3)	1.8 ± 0.2	1.9 (0.2)	1.8 ± 0.2	1.7 (0.3)	*t* = −1.74 (0.086)
Systolic BP (mm Hg)	132.2 ± 15	130.5 (23)	144.6 ± 10	145 (9.8)	119.8 ± 6.5	122 (9.2)	U = 9.5 (<0.001) **
Diastolic BP (mm Hg)	82.7 ± 7.9	83.5 (8.2)	87 ± 7.8	87 (7.2)	78.4 ± 5.3	79 (9)	U = 178 (<0.001) **
HR (beats/min)	72.4 ± 13.9	71 (17)	74.2 ± 15.7	69.5 (21.5)	70.6 ± 11.7	72 (14.5)	*t* = −1.098 (0.276)

BMI: body-mass index; BSA: body surface area; HR: heart rate; MV: mean value; SD: standard deviation; M: median; IQR: interquartile range; U: Mann–Whitney U test; *t*: Student *t*-test; ** *p* < 0.001.

**Table 2:** Parameters of cardiac morphology and function.

	All (N = 72)		Hypertensive (N = 36)		Normotensive (N = 36)		
Variable	MV ± SD	M (IQR)	MV ± SD	M (IQR)	MV ± SD	M (IQR)	*p* Value
HR (beats/min)	72.4 ± 13.9	71 (17)	74.2 ± 15.7	69.5 (21.5)	70.6 ± 11.7	72 (14.5)	*t* = −1.098 (0.276)
EATT (cm)	0.6 ± 0.3	0.6 (0.4)	0.8 ± 0.3	0.8 (0.3)	0.5 ± 0.1	0.4 (0.1)	U = 65.5 (<0.001) **
EATV (cm^3^)	13.7 ± 3.3	13.5 (5.5)	16.5 ± 1.9	16.4 (2.2)	10.9 ± 1.5	10.9 (2.5)	*t* = −13.815 (<0.001) **
LV-EF (%)	57.7 ± 4	57.8 (5.4)	58.5 ± 4	58.7 (4.2)	56.8 ± 3.9	56.8 (6.3)	*t* = −1.727 (0.089)
MM average (g/m^2^)	68 ± 14.2	66.2 (18.4)	68.6 ± 14.9	68.7 (21.2)	67.4 ± 13.6	65.5 (14.1)	*t* = −0.351 (0.726)
EDV (mL/m^2^)	90.9 ± 13	90.1 (19.1)	90 ± 14.8	89.8 (20.2)	91.9 ± 11.1	91.3 (16.4)	*t* = 0.622 (0.536)
ESV (mL/m^2^)	39.7 ± 8.2	38.4 (11.6)	38.9 ± 8.8	36.8 (11.5)	40.6 ± 7.6	39.6 (11)	U = 749.5 (0.255)
SV (mL/m^2^)	51.3 ± 8.6	51.2 (10.6)	51.8 ± 9.8	51.6 (1.,2)	50.7 ± 7.4	51 (8)	*t* = −0.528 (0.599)
Peak Filling Rate (mL/s/m^2^)	297.6 ± 89.8	290.1 (72.7)	303.8 ± 88.5	290.3 (77.2)	291.3 ± 91.9	289.5 (57.5)	U = 601 (0.603)
Peak Filling Time (ms)	421 ± 35.7	424.7 (36.7)	418.9 ± 32.5	422.9 (38.1)	423 ± 38.9	427.3 (36.6)	*t* = 0.485 (0.629)
Myocardial thickness LV (mm)	7.9 ± 1.3	7.8 (1.7)	8.1 ± 1.4	8.1 (1.7)	7.7 ± 1.1	7.7 (1.5)	*t* = −1.563 (0.123)

EATT: epicardial adipose tissue thickness; EATV: epicardial adipose tissue volume; IMT: intima-media thickness; LV-EF: left ventricular ejection fraction; MM average: average myocardial mass; EDV: end diastolic volume; ESV: end systolic volume; SV: stroke volume; MV: mean value; SD: standard deviation; M: median; IQR: interquartile range; U: Mann–Whitney U test; *t*: Student’s *t*-test; **: *p* < 0.001.

**Table 3:** Predictors of hypertension.

Predictor	AUC (95% CI)	Threshold	Sen	Spec	PPV	NPV
EATV	0.992 (0.981, 0.992)	13.545	94.4%	97.2%	97.1%	94.6%
EATT	0.949 (0.899, 0.949)	0.545	91.7%	88.9%	89.2%	91.4%

## Data Availability

The data sets generated and analyzed during the current study are available from the corresponding author upon reasonable request.
